# Oleanolic acid acrylate elicits antidepressant-like effect mediated by 5-HT_1A_ receptor

**DOI:** 10.1038/srep11582

**Published:** 2015-07-22

**Authors:** James O. Fajemiroye, Prabhakar R. Polepally, Narayan D. Chaurasiya, Babu L. Tekwani, Jordan K. Zjawiony, Elson A. Costa

**Affiliations:** 1 Department of Pharmacology, Federal University of Goias, Campus Samambaia, 74001-970, Goiania, GO, Brazil; 2 Department of BioMolecular Sciences, Division of Pharmacognosy, School of Pharmacy, University of Mississippi, P.O. Box 1848, University, MS 38677, USA; 3 National Center for Natural Products Research, University of Mississippi, P.O. Box 1848, University, MS 38677, USA; 4 Department of BioMolecular Sciences, Division of Pharmacology School of Pharmacy, University of Mississippi, P.O. Box 1848, University, MS 38677, USA

## Abstract

The development of new drugs for the treatment of depression is strategic to achieving clinical needs of patients. This study evaluates antidepressant-like effect and neural mechanisms of four oleanolic acid derivatives i.e. acrylate (D1), methacrylate (D2), methyl fumarate (D3) and ethyl fumarate (D4). All derivatives were obtained by simple one-step esterification of oleanolic acid prior to pharmacological screening in the forced swimming (FS) and open field (OF) tests. Pharmacological tools like α-methyl-*p*-tyrosine (AMPT, catecholamine depletor), *p*-chlorophenylalanine (serotonin depletor), prazosin (PRAZ, selective α1-receptor antagonist), WAY-100635 (selective serotonin 5-HT_1A_ receptor antagonist) as well as monoamine oxidase (MAO) and functional binding assays were conducted to investigate possible neural mechanisms. In the FS test, D1 showed the most promising antidepressant-like effect without eliciting locomotor incoordination. Unlike group of mice pretreated with AMPT 100 mg/kg, PCPA 100 mg/kg or PRAZ 1 mg/kg, the effect of D1 was attenuated by WAY-100635 0.3 mg/kg pretreatment. D1 demonstrated moderate inhibition of MAO-A (IC_50_ = 48.848 ± 1.935 μM), potency (pEC_50_ = 6.1 ± 0.1) and intrinsic activity (E_max_ = 26 ± 2.0%) on 5-HT_1A_ receptor. In conclusion, our findings showed antidepressant-like effect of D1 and possible involvement of 5-HT_1A_ receptor.

Depression is a chronic psychiatric disorder that contributes substantially to mental impairment, physical disability and socioeconomic burden^1^. This disorder is capable of reducing quality of life, productivity, and increase suicidality. World Health Organization predicts, depression to become the second leading cause of disease by 2020^2^,^3^. The symptoms of depression such as anhedonia, irritability, difficulties in concentrating and sleep are clear evidences of the complexity of its neurobiology^4^. The search for new drugs remains a desirable approach as the current agents including tricyclic antidepressants, selective serotonin reuptake and monoamine oxidase (MAO) inhibitors are still far from producing optimal effects without causing side effects^5^,^6^,^7^.

Preclinical and clinical studies of some medicinal plants such as *Cimicifuga foetida, Hypericum perforatum*, *Pimenta pseudocaryophyllus* among others have been reported to possess antidepressant property^8^,^9^,^10^,^11^,^12^. Oleanolic acid (OA), major active secondary metabolite isolated from *Pimenta pseudocaryophyllus,* has demonstrated numerous pharmacological activities such as hepatoprotection^13^,^14^, cytoprotection^15^, anti-oxidation^16^,^17^,^18^,^19^, anti-inflammation^20^, anti-cancer^21^,^22^, anti-ulcer^23^,^24^ and antidepressant^7^,^25^.

The diverse effects of OA are associated with its plurality of mechanism of action. The suppression of the inflammatory response by OA involves inhibition of C3-convertase^26^,^27^, reduction of prostaglandin PGE2 biosynthesis^28^ and exudates^29^. The hepatoprotective effect of OA involves inhibition of CYPB5, CYP1A and CYP2A, an increase in antioxidant substances such as glutathione, metallothionein, zinc, glutathione-S-transferase and glucuronosyltransferase^13^,^30^,^31^. Antidepressant-like effect of OA was found to be attenuated by depletion of indolamine and catecholamine^7^. However, unsuspecting health risks that are associated with the multiple interaction of OA constitute limitations to its therapeutic application. Relatively high availability of OA from plants offers immense opportunity for the synthesis and evaluation of its derivatives as potential antidepressant drugs. Previous biological studies of OA derivatives have shown their pharmacological potential^32^,^33^,^34^.

Herein we report the antidepressant-like effect of four new OA derivatives obtained by simple esterification reaction at C-3 with corresponding acyl chlorides.

## Results

### ^1^H, ^13^C NMR and MS data for compounds D1-D4

NMR and MS data were obtained for all products purified by column chromatography.

Oleanolic acid acrylate (D1): White amorphous powder, yield 90%. ^1^H NMR (400 MHz, CDCl_3_): δ 6.44 (d, *J* = 16.4 Hz, 1H), 6.16 (dd, *J* = 16.4, 7.6 Hz, 1H), 5.85 (d, *J* = 7.6 Hz, 1H), 5.29 (dd, *J* = 5.8, 2.6 Hz, 1H), 4.53 (dd, *J* = 9.4, 6.2 Hz, 1H), 2.81 (dd, *J* = 14.2, 4.0 Hz, 1H), 1.86 (m, 1H), 1.74 (m, 1H), 1.65–1.54 (m, 6H), 1.44 (m, 1H), 1.38–1.27 (m, 3H), 1.22 (m, 1H), 1.15 (m, 1H), 1.14 (s, 3H), 1.07–0.97 (m, 7H), 0.96 (s, 3H), 0.93 (m, 1H), 0.92 (s, 3H), 0.90 (s, 3H), 0.88 (s, 3H), 0.87 (s, 3H), 0.85 (s, 3H), 0.76 (s, 3H). ^13^C NMR (100 MHz, CDCl_3_): δ 185.1, 167.3, 144.7, 131.5, 129.3, 122.7, 80.3, 56.1, 47.9, 46.8, 46.1, 42.2, 41.1, 40.2, 38.3, 37.2, 33.9, 33.1, 32.7, 32.4, 30.6, 30.1, 29.2, 27.9, 26.2, 23.8, 23.1, 22.9, 18.4, 18.1, 17.4, 16.9, 15.7; HRESIMS (*m/z*): [M + H]^+^ calculated for C_33_H_50_O_4_, 511.3709; found, 511.3712.

Oleanolic acid methacrylate (D2): White amorphous powder, 83% yield. ^1^H NMR (400 MHz, CDCl_3_): δ 6.51 (s, 1H), 6.43 (s, 1H), 5.31 (dd, *J* = 5.8, 2.6 Hz, 1H), 4.55 (dd, *J* = 9.4, 6.2 Hz, 1H), 2.83 (dd, *J* = 14.2, 4.0 Hz, 1H), 1.99 (s, 3H), 1.85 (m, 1H), 1.73 (m, 1H), 1.66–1.53 (m, 6H), 1.45 (m, 1H), 1.37–1.26 (m, 3H), 1.21 (m, 1H), 1.16 (m, 1H), 1.13 (s, 3H), 1.06–0.98 (m, 7H), 0.96 (s, 3H), 0.94 (m, 1H), 0.92 (s, 3H), 0.89 (s, 3H), 0.88 (s, 3H), 0.87 (s, 3H), 0.86 (s, 3H), 0.77 (s, 3H). ^13^C NMR (100 MHz, CDCl_3_): δ 185.7, 167.5, 144.3, 136.6, 131.8, 129.6, 125.7, 122.9, 80.4, 56.2, 47.8, 46.9, 46.3, 42.4, 41.2, 40.4, 38.5, 37.3, 33.9, 33.2, 32.8, 32.5, 30.8, 30.2, 29.3, 27.8, 26.3, 23.9, 23.2, 23.0, 18.5, 18.2, 17.8, 17.5, 16.8, 15.9; HRESIMS (*m/z*): [M + H]^+^ calculated for C_34_H_52_O_4_, 525.3866; found, 525.3871.

Oleanolic acid methyl fumarate (D3): White amorphous powder, 75% yield. ^1^H NMR (400 MHz, CDCl_3_): δ 6.29 (s, 2H), 5.28 (dd, *J* = 5.8, 2.6 Hz, 1H), 4.56 (dd, *J* = 9.4, 6.2 Hz, 1H), 3.81 (s, 3H), 2.84 (dd, *J* = 14.2, 4.0 Hz, 1H), 1.87 (m, 1H), 1.75 (m, 1H), 1.64-1.52 (m, 6H), 1.45 (m, 1H), 1.38–1.26 (m, 3H), 1.23 (m, 1H), 1.16 (m, 1H), 1.15 (s, 3H), 1.08–0.97 (m, 7H), 0.96 (s, 3H), 0.93 (m, 1H), 0.92 (s, 3H), 0.90 (s, 3H), 0.88 (s, 3H), 0.86 (s, 3H), 0.85 (s, 3H), 0.75 (s, 3H). ^13^C NMR (100 MHz, CDCl_3_): δ 185.1, 168.3, 167.1, 144.7, 133.8, 130.1, 122.9, 80.5, 56.3, 52.3, 47.6, 46.9, 46.3, 42.8, 41.3, 40.6, 38.8, 37.5, 33.8, 33.1, 32.5, 32.4, 30.6, 30.1, 29.2, 27.9, 26.6, 23.8, 23.2, 22.8, 18.5, 18.2, 17.6, 16.3, 15.5; HRESIMS (*m/z*): [M + H]^+^ calculated for C_35_H_52_O_6_, 569.3764; found, 569.3769.

Oleanolic acid ethyl fumarate (D4): White amorphous powder, 80% yield. ^1^H NMR (400 MHz, CDCl_3_): δ 6.31 (s, 2H), 5.28 (dd, *J* = 5.8, 2.6 Hz, 1H), 4.58 (dd, *J* = 9.4, 6.2 Hz, 1H), 3.89 (q, 2H), 2.83 (dd, *J* = 14.2, 4.0 Hz, 1H), 1.89 (m, 1H), 1.76 (m, 1H), 1.65–1.53 (m, 6H), 1.45 (m, 1H), 1.38–1.26 (m, 3H), 1.23 (m, 1H), 1.21 (t, 3H), 1.16 (m, 1H), 1.15 (s, 3H), 1.08–0.97 (m, 7H), 0.96 (s, 3H), 0.93 (m, 1H), 0.92 (s, 3H), 0.90 (s, 3H), 0.88 (s, 3H), 0.86 (s, 3H), 0.85 (s, 3H), 0.75 (s, 3H). ^13^C NMR (100 MHz, CDCl_3_): δ 185.4, 168.7, 167.3, 144.8, 132.9, 130.1, 122.9, 80.5, 61.5, 56.3, 47.6, 46.9, 46.3, 42.8, 41.3, 40.7, 38.9, 37.4, 33.8, 33.3, 32.5, 32.5, 30.8, 30.1, 29.2, 27.9, 26.6, 23.8, 23.2, 22.8, 18.5, 18.1, 17.8, 16.5, 15.3, 14.4; HRESIMS (*m/z*): [M + H]^+^ calculated for C_36_H_54_O_6_, 583.3921; found, 583.3927.

### Effects of D1 treatment in the forced swimming (FS) and open field (OF) tests

**Figure 1:**
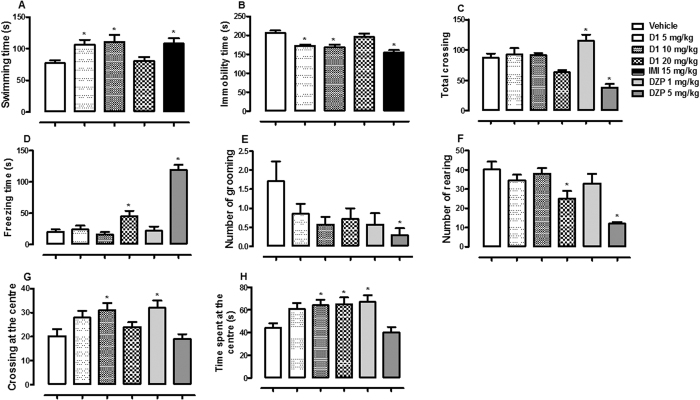
The effect of oral administration of vehicle 10 mL/kg, acrylate (D1) 5, 10, 20 mg/kg or imipramine (IMI) 15 mg/kg on the swimming (**A**) and immobility time in the FS test (**B**). Figure 1 C, D, E, F, G and H showed the effect of oral administrations of vehicle 10 mL/kg, D1 5, 10, 20 mg/kg, diazepam (DZP) 1 or 5 mg/kg on the total crossing, freezing time, number of grooming, number of rearing, crossing at the centre, and time spent at the centre of the OF by mice, respectively. Each column represents the mean ± SEM of 10 animals. *p < 0.05 vs vehicle treated group.

Oral administration of D1 elicited significant effect on swimming [F (4, 30) = 4.28, p < 0.01, Fig. 1A] and immobility time [F (4, 30) = 10.34, p < 0.001, Fig. 1B]. Dunnett´s test showed significant increase in swimming time by D1 5 or 10 mg/kg (p < 0.05) and reduction in immobility time at the same doses (p < 0.05). In the OF test, D1 and/or DZP elicited significant effects on total crossing [F (5, 36) = 13.60, p < 0.001, Fig. 1C], freezing time [F (5, 36) = 36.51, p < 0.001, Fig. 1D], number of grooming [F (5, 36) = 2.49, p < 0.05, Fig. 1E], number of rearing [F (5, 36) = 8.55, p < 0.001, Fig. 1F], crossing at the centre [F (5, 36) = 4.24, p < 0.01, Fig. 1G] and time spent at the centre [F (5, 36) = 5.10, p < 0.01, Fig. 1H] of OF. D1 elicited an increase in freezing time at dose 20 mg/kg (p < 0.05), crossing at the centre at dose 10 mg/kg (p < 0.05) and time spent at the centre at doses 10 and 20 mg/kg (p < 0.05), and a reduction in the number of grooming at dose 20 mg/kg (p < 0.05). However, other parameters were not altered by D1 (p > 0.05).

### Effects of D2 treatment in the FS and OF tests

**Figure 2:**
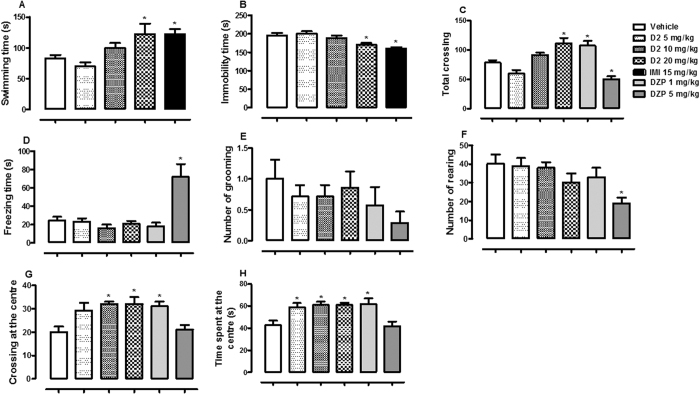
The effect of oral administration of vehicle 10 mL/kg, methacrylate (D2) 5, 10, 20 mg/kg or imipramine (IMI) 15 mg/kg on the swimming (**A**) and immobility time in the FS test (**B**). Figure 2 C, D, E, F, G and H showed the effect of oral administrations of vehicle 10 mL/kg, D2 5, 10, 20 mg/kg, diazepam (DZP) 1 or 5 mg/kg on the total crossing, freezing time, number of grooming, number of rearing, crossing at the centrre, and time spent at the centre of the OF by mice, respectively. Each column represents the mean ± SEM of 10 animals. *p < 0.05 vs vehicle treated group.

D2 elicited significant effect on swimming [F (4, 30) = 5.59, p < 0.01, Fig. 2A] and immobility time [F (4, 30) = 9.27, p < 0.001, Fig. 2B]. Dunnett´s test showed significant increase in swimming time and reduction in immobility time by D2 20 mg/kg (p < 0.05). Except for number of grooming [F (5, 36) = 1.03, p > 0.05, Fig. 2E] in the OF test, D2 and/or DZP showed significant effects on total crossing [F (5, 36) = 15.34, p < 0.001, Fig. 2C], freezing time [F (5, 36) = 10.24, p < 0.001, Fig. 2D], number of rearing [F (5, 36) = 3.39, p < 0.05, Fig. 2F], crossing at the centre [F (5, 36) = 5.35, p < 0.001, Fig. 2G] and time spent at the centre [F (5, 36) = 6.26, p < 0.001, Fig. 2H] of OF. D2 induced an increase in total crossing (at dose 20 mg/kg), crossing at the centre (at doses 10 and 20 mg/kg) and time spent at the centre (at doses 5, 10 and 20 mg/kg). However, other parameters were not altered by D2 (p > 0.05).

### Effects of D3 treatment in the FS and OF tests

**Figure 3:**
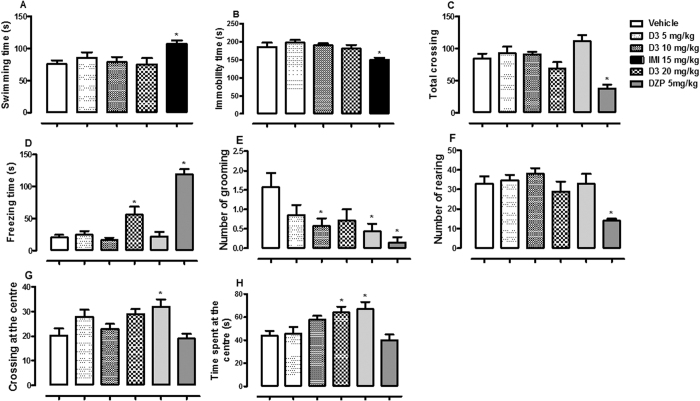
The effect of oral administration of vehicle 10 mL/kg, methyl fumarate (D3) 5, 10, 20 mg/kg or imipramine (IMI) 15 mg/kg on the swimming (**A**) and immobility time in the FS test (**B**). Figure 3 C, D, E, F, G and H showed the effect of oral administrations of vehicle 10 mL/kg, D3 5, 10, 20 mg/kg, diazepam (DZP) 1 or 5 mg/kg on the total crossing, freezing time, number of grooming, number of rearing, crossing at the centre and time spent at the centre of the OF by mice, respectively. Each column represents the mean ± SEM of 10 animals. *p < 0.05 vs vehicle treated group.

Oral administration of D3 did not elicit significant effect on swimming [F (4, 30) = 3.04, p > 0.05, Fig. 3A] and immobility time [F (4, 30) = 4.72, p > 0.05, Fig. 3B]. In the OF test, D3 and/or DZP showed significant effects on total crossing [F (5, 36) = 9.78, p < 0.001, Fig. 3C], freezing time [F (5, 36) = 28.99, p < 0.001, Fig. 3D], number of grooming [F (5, 36) = 3.67, p < 0.01, Fig. 3E], number of rearing [F (5, 36) = 5.23, p < 0.01, Fig. 3F], crossing at the centre [F (5, 36) = 4.35, p < 0.01, Fig. 3G] and time spent at the centre [F (5, 36) = 5.28, p < 0.01, Fig. 3H] of OF. D3 induced an increase in freezing time (at dose 20 mg/kg), time spent at the centre (at dose 20 mg/kg) and a reduction in the number of grooming (at dose 10 mg/kg). However, other parameters were not altered by D3 (p > 0.05).

### Effects of D4 treatment in the FS and OF tests

**Figure 4:**
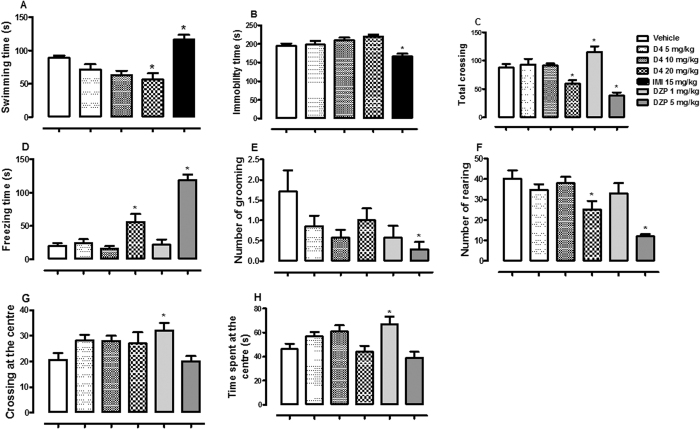
The effect of oral administration of vehicle 10 mL/kg, ethyl fumarate (D4) 5, 10, 20 mg/kg or imipramine (IMI) 15 mg/kg on the swimming (**A**) and immobility time in the FS test (**B**). Figure 3 C, D, E, F, G and H showed the effect of oral administrations of vehicle 10 mL/kg, D4 5, 10, 20 mg/kg, diazepam (DZP) 1 or 5 mg/kg on the total crossing, freezing time, number of grooming, number of rearing, crossing at the centre, and time spent at the centre of the OF by mice, respectively. Each column represents the mean ± SEM of 10 animals. *p < 0.05 vs vehicle treated group.

D4 elicited significant effect on swimming [F (4, 30) = 11.11, p < 0.001, Fig. 4A] without changes in immobility time [F (4, 30) = 7.35, p > 0.05, Fig. 4B]. Dunnett´s test showed significant reduction in swimming time by D4 20 mg/kg (p < 0.05). In the OF, statistical analysis showed the significant effects of D4 and/or DZP on total crossing [F (5, 36) = 13.36, p < 0.001, Fig. 4C], freezing time [F (5, 36) = 28.67, p < 0.001, Fig. 4D], number of grooming [F (5, 36) = 2.51, p < 0.05, Fig. 4E], number of rearing [F (5, 36) = 8.31, p < 0.001, Fig. 4F], crossing at the centre [F (5, 36) = 2.78, p < 0.05, Fig. 4G] and time spent at the centre [F (5, 36) = 4.98, p < 0.001, Fig. 4H] of OF. D4 induced a reduction in number of rearing, total crossing and an increase in freezing time at dose 20 mg/kg (p < 0.05).

### Effects of AMPT, PCPA PRAZ or WAY pretreatments on antidepressant-like property of D1

**Figure 5:**
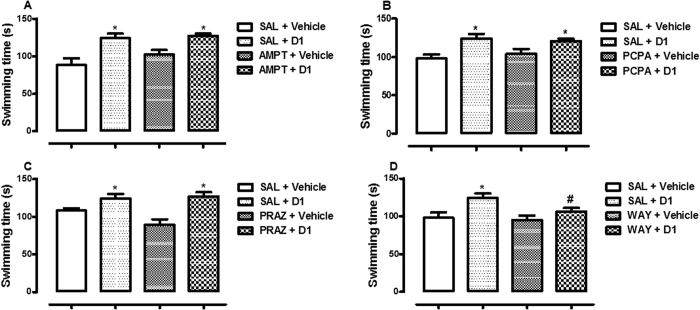
Alteration in swimming time following pretreatment with saline solution (SAL) 10 mL/kg or *α*-methyl-p-tyrosine (AMPT) 100 mg/kg, Fig. 5A; SAL 10 mL/kg or *p*-chlorophenylalanine methyl ester (PCPA) 100 mg/kg, Fig. 5B; SAL 10 mL/kg or prazosin (PRAZ) 1 mg/kg, Fig. 5C; SAL 10 mL/kg or WAY-100635 (WAY) 0.3 mg/kg, Fig. 5D prior to oral treatment with vehicle 10 mL/kg or acrylate (D1) 5 mg/kg. Data are expressed as mean ± SEM, n = 5; *p < 0.05 vehicle treated group versus other experimental groups; # p < 0.05 SAL + D1 versus WAY + D1.

Figure 5 showed the effect of pretreatment (AMPT, PCPA, PRAZ or WAY; independent variable) and treatment (vehicle or D1; independent variable) on the swimming time (dependent variable and main effect) in the FS test. The data obtained on the main effect did not revealed interaction between the independent variables (p > 0.05). Pairwise comparisons with Bonferroni post hoc test showed that D1 administration increased the swimming time in the Fig. 5A–D (i.e. SAL + Vehicle versus SAL + D1, p < 0.05). Pretreatment with AMPT did not attenuate antidepressant-like effect of D1 (i.e. SAL + D1 versus AMPT + D1, p > 0.05, Fig. 5A). In addition, pretreatment with PCPA and PRAZ did not block antidepressant-like effect of D1 (i.e. SAL + D1 versus PCPA + D1, p > 0.05, Fig. 5B) and (SAL + D1 versus PRAZ + D1, p > 0.05, Fig. 5C), respectively. In contrary, WAY pretreatment induced a significant blockade of the antidepressant-like effect of D1 (i.e. SAL + D1 versus WAY + D1; p < 0.05, Fig. 5D). Pretreatments with AMPT, PCPA, PRAZ or WAY, prior to vehicle administration did not alter animal behaviour at the doses tested (i.e AMPT + Vehicle, PCPA + Vehicle, PRAZ + Vehicle, WAY + Vehicle versus SAL + Vehicle; p > 0.05).

### Effects of D1 on enzymatic activity of MAO-A and -B

D1 showed an IC_50_ of 48.848 ± 1.935 μM for MAO-A (moderate inhibition) and IC_50_ value >100 μM for MAO-B. The reference compounds clorgyline and deprenyl showed potent inhibition of MAO-A and -B with IC_50_ of 0.003 ± 0.0001 and 0.047 ± 0.0041 μM, respectively.

### Functional activity of D1 at 5-HT_1A_ receptor

Unlike WAY, D1 produced an increase in [^35^S]GTPγS binding to mice hippocampal membranes. Potency and intrinsic activity of D1 on 5-HT_1A_ receptor were close to that of buspirone (Table 1).

### General pharmacological test of D1 effect on mice

In the general pharmacological test, D1 elicited a reduction in exploratory activity at the dose of 250 mg/kg after 30 minutes of subcutaneous (s.c) administration (Table 2). A reduction in exploratory activity and sedative effect were observed following intraperitoneal (i.p) and s.c administration of D1 at doses 10, 50 and 250 mg/kg between 30 minute and 1hour. Oral (p.o) administration of D1 elicited increase in exploratory activity starting from 30 minute to 1 hour at the doses of 10, 50 and 250 mg/kg (Table 2). All the behavioural manifestations disappeared within 4 hours of D1 administration without any record of toxicity or death.

## Discussion

Chemical modification remains an important strategy for new drug synthesis and development from natural products^35^. The rationale for modification of oleanolic acid structure by esterification with acrylic and fumaric acid chlorides was to introduce electrophilic centre through the formation of Michael acceptor-type OAD that could interact with nucleophilic residues of binding site of the receptor.

In order to investigate antidepressant-like effects, mice were treated orally with OAD and subjected to the FS test. The FS test is one of the most used behavioural tests for screening antidepressant-like activity of new compounds^36^,^37^. Oral administration of D1 or D2 increased the swimming time. In contrary, D4 reduced this parameter while D3 did not show any significant effect on this parameter. An increase in swimming time by D1 or D2 administration that was accompanied by a significant reduction in immobility time is a valid measure of antidepressant-like effect in this model.

The sensitivity of FS test to acute antidepressant treatment, stimulants, sedatives, myorelaxants or motor-impairing compounds can influence animal performance in this model and lead to false-negative or false-positive results^38^. Hence, it is imperative to assess locomotor activity of the mice in the OF test in order to ascertain that the antidepressant-like effect of OAD was not masked by other general pharmacological activity. An open field test provides simultaneous measurement of locomotion, exploratory and anxiolytic activities^39^. The effects of D4 in the FS (reduction of swimming time) and OF (increase in freezing time, decrease in total crossing and number of grooming activities) could be associated to a sedative or myorelaxant effect of this derivative. In contrary, a dose dependent increase in swimming time and reduction in immobility time in the FS test by D1 without any alteration in total crossing, grooming activity and freezing time in the OF test reinforced specificity of antidepressant-like effect of D1. The increase in total crossing (an effect that could suggests stimulation) by D2 20 mg/kg (a dose that induced increase in swimming time and reduction in immobility time) makes further investigation of D1 more promising. In addition to antidepressant-like effect, an increase in crossing and time spent at the centre of OF suggests anxiolytic-like effect of D1 at the dose of 10 mg/kg. Anti-anxiety drugs like diazepam (benzodiazepine receptor agonist) or buspirone (partial agonist of 5-HT_1A_ receptor) are known to alter these parameters^40^. The choice of reference drugs and their respective doses in the FS and OF tests was based on previous study^7^. The IMI 15 mg/kg elicited antidepressant-like effect in FS test without altering locomotor activity of mice in the OF test while the DZP 1 or 5 mg/kg induced anxiolytic and sedative like effect, respectively.

Having demonstrated antidepressant-like effect of D1 at the dose of 5 mg/kg, pharmacological tools like AMPT and PCPA (tyrosine and tryptophan hydroxylase enzyme inhibitors, respectively), PRAZ and WAY-100635 (selective antagonist of α1- adrenoceptor and 5HT_1A_ receptors, respectively) were used to investigate possible mechanism of antidepressant-like effect of D1. The modulation of monoamine neurotransmission, transport and/or inhibition of their oxidative deamination by MAO have been associated with the mechanism of action of many clinically available antidepressant drugs^41^. In the present study, depletion of serotonin with PCPA or catecholamine with AMPT did not attenuate the antidepressant-like effect of D1. Unlike PRAZ, WAY-100635 pretreatment blocked the effect of D1, thereby suggesting involvement of 5HT_1A_ receptor. Unlike previous study that showed the blockade of antidepressant-like effect of OA by PCPA, AMPT, NAN, PRAZ and WAY100635^7^, the effects of D1 seems to be devoid of plural mechanisms.

The inhibition and/or activation of metabolic enzymes that are involved in the synthesis or breakdown of monoamine are critical targets of some antidepressant drugs. Although selective inhibitors of MAO-B are clinically use for the treatment of neurological disorders such as Parkinson’s disease and Alzheimer’s disease, the isoenzyme MAO-A inhibitors are currently in use for the treatment of depression^42^. MAO is an outer mitochondrial membrane bound flavin adenine dinucleotide-linked enzyme involved in the oxidative deamination of biogenic amines and xenobiotic arylalkylamines to aldehydes^43^. Serotonin (5-HT) and norepinephrine are preferentially metabolized by MAO-A isoform whereas MAO-B isoform deaminates benzylamine as a substrate. Previous study showed that antidepressant-like effect of OA was independent of MAO activity and that the levels of 4-hydroxy-3-methoxyphenylglycol and 3,4-dihydroxyphenylacetic acid in the brain hippocampus and cortex remained unaltered after administration of OA^7^,^25^. Consistent with these results, D1 showed only moderate inhibitory effect on MAO-A and no effect on MAO B. Unlike D1, clorgyline and deprenyl showed significant inhibition of MAO isoenzymes. Clorgyline and (*R*)-deprenyl have been reported to be potent and selective inhibitors of MAO-A and -B, repectively^42^,^44^.

Although the antidepressant-like effect of D1 could be associated to the inhibition of MAO A, the preservation of antidepressant-like effect of D1 despite AMPT and PCPA pretreatments suggests that the enzymes that are involved in the synthesis of monoamines (tyrosine and tryptophan hydroxylase, respectively) are possibly not critical to the effect of D1. Hence, we hypothesized that the antidepressant-like effect of the acute oral administration of this new oleanolic acid derivative is independent of monoamine synthesis. The inhibition of MAO A by D1 could increase synaptic concentration of 5-HT, bioavailability, prolongation of its interaction with 5-HT_1A_ receptor and subsequent potentiation of serotonergic transmission. The blockade of D1 effect by WAY-100635, which seems to be consistent with serotonergic mechanism, is not sufficient to discriminate between direct activity of D1 on the 5-HT_1A_ receptor or indirect effect through inhibition of MAO A to facilitate high level of 5-HT.

The interaction of 5-HT with diverse serotonergic receptors has been hypothesized in the regulation of neuronal activity^45^. As 5-HT_1A_ receptor seems to be involved in the effect of D1, a functional characterization of D1 at this serotonergic receptor subtype *in vitro* became necessary. Our findings showed similar potency and intrinsic activity of D1 and buspirone. The variations in the intrinsic activity could differentiate among full agonists, partial agonists, and antagonists, based on their high, intermediate, and zero intrinsic activity, respectively^46^. Unlike (+)8-OH-DPAT which is full agonist of 5-HT_1A_ receptors, D1 could be considered as a partial agonist of this receptor on the basis of its the intrinsic activity. The 5-HT_1A_ receptors are relevant to the clinical response to antidepressant drugs. They are located presynaptically in the raphe nuclei (where they act as cell body autoreceptors to inhibit serotonergic transmision) and postsynaptically in limbic and cortical regions to attenuate firing activity^47^. The azapirones are full agonists at 5-HT_1A_ autoreceptors and are generally partial agonists at postsynaptic 5-HT_1A_ receptors (e.g buspirone).

In addition to the promising therapeutic value of D1, the data on general pharmacological test showed that this OAD did not elicit any sign of toxicity or behavioural alterations that could constitute harm to the mice even at the highest dose of 250 mg/kg. Except for the mild sedation that was observed within 4 hours of the administration at the highest dose, there was no record of weight gain or animal’s death during the 7 days of observation. These observations demonstrate that the chemical modification of OA did not make the derivative unsafe for administration.

In conclusion, four new Michael acceptor-type oleanolic acid derivatives esterified on C-3 were synthesized and their antidepressant-like activity investigated. It is important to reiterate that the therapeutic application of OA or its derivatives is still very limited due to dearth of pharmacological data. The overall findings showed promising antidepressant-like property of D1. The mechanism of antidepressant-like effect of this compound suggested the involvement of 5HT_1A_ receptor.

## Methods

### Experimental animals

Male Swiss mice (weighing between 25–30 g; 6–8 weeks old), provided by Central Animal House of the Federal University of Goiás, were used in all behavioural models. The experimental animals were kept in an intra-laboratory facility cage (32 × 18 × 16 cm) under controlled environmental conditions (23 ± 1 °C, 12 hr light-dark cycle) with access to food and water *ad libitum*. The experimental protocols (number 104/08) were approved by Ethical Committee of Federal University of Goiás in accordance to the international laws on the care and use of laboratory animals.

### Drugs and Treatment

Oleanolic Acid (OA), kynuramine, clorgyline, deprenyl, buspirone, Tween 80 (2% polyoxyethylenesorbitan monooleate), *p*-chlorophenylalanine (PCPA), N-{2-[4-(2-methoxyphenyl)-1-piperazinyl]ethyl}-N-2-pyridinylcyclohexane carboxamide (WAY-100635 or WAY), Tris-HCl, ethylenediaminetetraacetic acid (EDTA), bovine serum albumin (BSA), polyethylenimine, 8-OH-DPAT, [^35^S]-GTPγS and dimethyl Sulfoxide (DMSO) were purchased from Sigma-Aldrich, St Louis, MO, USA. Diazepam (DZP), imipramine (IMI) and prazosin (PRAZ) were purchased from Cristália, Itapira, SP, Brazil. Recombinant Human Monoamine Oxidase-A and -B (MAO-A and -B) were purchased from BD Biosciences Bedford, MA, USA. Oleanolic acid derivatives (OAD); oleanolic acid acrylate (D1), oleanolic acid methacrylate (D2), oleanolic acid methyl fumarate (D3) and oleanolic acid ethyl fumarate (D4) were synthesized by esterification of OA with corresponding acyl chlorides. For *in vivo* assay, drugs were dissolved in a vehicle [a mixture of 0.9% NaCl and 5% Tween-80 (v/v)] and administered orally (p.o.) or intraperitonealy (i.p) in a volume of 0.1 mL per 10 g of mice body weight. All control animals received vehicle on the same regimen as the treated groups. For *in vitro* assay, drugs were dissolved in DMSO to yield a final DMSO concentration of 1.0% in the reaction mixture.

### General Experimental Procedures:

All commercially available reagents were used without further purification. All these reactions were performed under Argon atmosphere and anhydrous dichloromethane was used as solvent, which was purchased from Sigma-Aldrich. The ^1^H NMR spectra were recorded on a Bruker Avance–400 spectrometer using CDCl_3_ as solvent, δ values in ppm and coupling constant *J* (Hz) assignments of ^1^H resonance coupling. Thin-layer chromatography (TLC) was performed on 250 μm layer plates Whatmann PE SIL G/UV silica gel (backing polyester) plates using *n*-hexane/EtOAc, 1:1 solvent system. Spots on TLC visualized with anisaldehyde / H_2_SO_4_ in methanol. Column chromatography was performed with silica gel (230 × 400 mesh) from GA, USA. Analytical HPLC was carried out on Waters 2487 with duel λ Absorbance detector system on Phenomenex Luna-C_18_ column (4.6 × 250 mm, 5 μm) with elution at a flow rate 1.0 mL/min. All biologically evaluated compounds were found to possess more than 95% purity by HPLC.

### Synthesis of OAD

**Figure 6:**
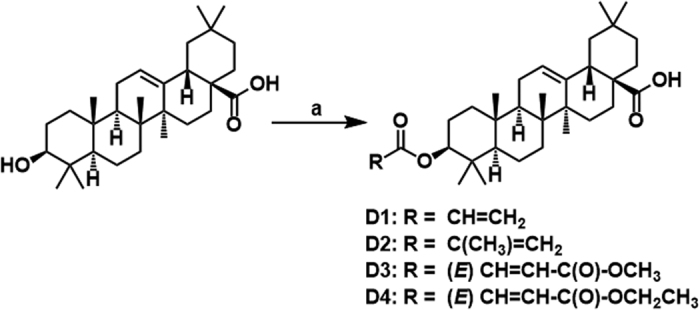
The synthetic scheme for the designed compounds D1-D4 with (a) reagents and conditions - appropriate acid chloride, Et_3_N, dry, DCM, N_2_, rt, 4 h.

General procedure for the synthesis of OAD (D1, D2, D3 and D4): Oleanolic acid (100 mg, 0.05 mmol) and a catalytic amount of trimethylamine were dissolved in dichloromethane (15 mL). An appropriate acid chloride (0.125 mmol) was added, and the reaction mixture was stirred for 4 h at room temperature. After TLC indicated completion of the reaction, the reaction mixture was quenched with water and the organic layer separated. The organic phase was washed with dilute aqueous HCl (0.01 mol/L, 2 mL) followed by saturated NaHCO_3_ (2 mL). The organic layer was dried over anhydrous MgSO_4_, evaporated, and the residue was purified by column chromatography (SiO_2_; eluent: CHCl_3_/MeOH) to obtain the target products with 75–90% yield. The synthetic scheme for the designed compounds D1-D4 is shown in Fig. 6 with (a) reagents and conditions - appropriate acid chloride, Et_3_N, dry.

### FS test

In the FS model, mice were randomly divided into 5 groups [control (vehicle), 3 different doses of OAD and IMI]. Mice were exposed to a modified version of FS test^48^ after oral administration of vehicle, OAD (5, 10 or 20 mg/kg) or IMI 15 mg/kg. FS apparatus is a cylindrical water container (42 cm in height, 18 cm in diameter) filled with water up to 30 cm (total volume (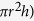 of 7635.06 cm^3^ at 24 ± 2 °C). In this model, the initial escape-directed behaviour like swimming and climbing by mouse are generally followed with a relatively passive and immobile posture. The container was cleaned with 10% ethanol solution prior to the exposure of naïve mouse. This test session (6-min duration) was videotaped and the swimming and immobility time was later scored and analyzed.

### OF test

In the OF, animals were randomly divided into six groups [control (vehicle), 3 different doses of OAD, DZP 1 or 5 mg/kg]. Animals were treated with vehicle, OAD (5, 10 or 20 mg/kg, p.o), DZP (1 or 5 mg/kg, p.o) as described in our previous work^7^,^49^, and placed in a circular open field [base area (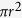) = 62.80 cm^2^ which is divided into 8 equal sectors enclosed in a 50 cm high wooden wall]. The test session (5-min duration) was videotaped in a sound-proof experimental room. Parameters like total crossing, freezing time, number of grooming and rearing activity, crossing at the centre and time spent at the centre of the open field were observed and scored.

### Mechanism of antidepressant-like effect of D1

Mice were pretreated (i.p) with saline solution or AMPT 100 mg/kg (catecholamine depletor) 4 h prior to the oral administration of vehicle 10 mL/kg or D1 5 mg/kg. It was demonstrated that AMPT (tyrosine hydroxylase inhibitor) reduced 57% of dopamine and 53% of noradrenaline levels in mice without affecting the levels of serotonin^50^. In a separate experiment, pretreatment (i.p) of mice with saline solution or PCPA 100 mg/kg (serotonin depletor) for four consecutive days prior to oral administration of vehicle or D1 5 mg/kg was used to investigate effect of serotonin depletion on antidepressant-like activity of this OAD. The regimen of PCPA in this study has been reported to deplete about 60% of endogenous serotonin content without altering the noradrenaline or dopamine levels^51^,^52^,^53^. Two separate experiment were also conducted where mice were pretreated with PRAZ (α1- adrenoceptor antagonist) 1 mg/kg or WAY-100635 - WAY (a selective antagonist of 5-HT_1A_) 0.3 mg/kg prior to the oral administration of vehicle 10 mL/kg or D1 5 mg/kg (30 minutes interval between i.p. pretreatment and p.o. treatment). In all the four experiments, oral administration of vehicle or D1 5 mg/kg was followed by FS test (60 min interval between p.o. treatment and behavioural testing).

### Inhibition assay using recombinant human MAO-A and -B

In order to investigate the effect of OAD on the activities of recombinant human MAO-A and -B, the kynuramine deamination assay was performed in 96-well plates as described earlier^54^. The assays were performed at a fixed concentration of the substrate (kynuramine) and varying inhibitor concentrations to determine the IC_50_ values. The substrate concentration of 80 and 50 μM kynuramine was chosen for MAO-A and -B, respectively, based on the previously reported apparent K_m_ values (the substrate concentration at half V_max_) for substrate binding^55^. The assay was performed with the addition of inhibitor. Inhibition was calculated as percent of product formation compared to the corresponding control (enzyme-substrate reaction) without the inhibitors. The reactions were carried out in 0.1 M potassium phosphate buffer at pH 7.4. Incubation mixtures contained 5 μg/mL of MAO-A (50 μL in buffer) or 12.5 μg/mL of MAO-B (50 μL in buffer). The compounds were dissolved in DMSO. The total reaction volume was 200 μL yielding a final DMSO concentration of 1.0% in the reaction mixture. The reaction mixtures were pre-incubated for 10 min at 37 °C followed by the addition of MAO-A or MAO-B to initiate the enzymatic reactions. The assay plates were incubated for 20 min at 37 °C and enzymatic reactions were stopped by the addition of 75 μL of 2N NaOH. The formation of 4-hydroxyquinoline was determined fluorometrically by SpectraMax M5 fluorescence plate reader (Molecular Devices, Sunnyvale, CA) with an excitation and emission wavelength of 320 nm and 380 nm, respectively, using the Soft Max Pro program.

### Binding of [^35^S]-GTPγS in mice hippocampus

The hippocampi of mice were dissected and homogenized in 5 volumes of ice-cold buffer (25 mM Tris-HCl, 0.5 mM dithiothreitol, and 0.5 mM EGTA, pH 7.4). The homogenate was centrifuged (1,000 × g for 5 min at 4 °C) and the resultant supernatant was removed. The remaining pellet was resuspended in buffer, homogenized, and centrifuged. The two supernatants obtained were combined and centrifuged at 1,000 × g for 20 min at 4 °C and the pellets were resuspended in membrane preparation buffer. The volumes were adjusted prior to the determination of protein concentration by Bradford method^56^ and stored at −80 °C until required. The (+)8-OH-DPAT, buspirone, WAY-100635 or D1 was incubated for 30 min at 30 °C with hippocampal membranes (approximately 10 μg protein) and 225 μl buffer (50 mM NaCl, 0.5 mM EGTA, 50 mM Tris–HCl, 0.5 mM MgCl_2_ and 50 μM GDP, 0.5 mM dithiothreitol, pH 7.5). The reactions were initiated by the addition of 25 μl [^35^S]-GTPγS, followed by a 20 min incubation at 20 °C. The reactions were terminated by rapid filtration. The filters were placed in liquid scintillation cocktail in counting vials. The liquid scintillation spectroscopy was used to measure the radioactivity bound to the filter paper after 10 hour.

### General pharmacological test

A modified method that was adopted by Malone^57^ was used in the present study to evaluate general pharmacological alterations in mice following administration of D1. This test permits the observation of behavioural change, report of possible toxic effect or death occurrence in addition to variations in pharmacological responses to different route of drug administrations. Group of animals were subjected to s.c, i.p, or p.o route of D1 administrations at the doses of 2, 10, 50 or 250 mg/kg or vehicle 10 mL/kg and observed for 7 days.

### Statistical analyses

Data were expressed as group mean ± S.E.M. A one-way analysis of variance (ANOVA) was used to detect behavioural changes elicited by drug treatment. Pairwise comparisons of individual treatment group to vehicle treated group were subsequently carried out with Dunnett´s test (post hoc test). A two-way ANOVA was used to detect the effect of treatment or pretreatment on the swimming time. This was followed up by pairwise comparisons of individual treatment group with Bonferroni test (post hoc test). The estimation of potency and efficacy were determined by nonlinear regression analysis using GraphPad Prism version 5.00 (GraphPad Software, San Diego, CA, USA). The p value of less than 0.05 was considered significant^58^.

## Additional Information

**How to cite this article**: Fajemiroye, J. O. *et al.* Oleanolic acid acrylate elicits antidepressant-like effect mediated by 5-HT_1A_ receptor. *Sci. Rep.* **5**, 11582; doi: 10.1038/srep11582 (2015).

## Figures and Tables

**Table 1:** The estimates of functional parameters (agonist potency and intrinsic activity) for D1 and standard compounds in a [^35^S]GTPγS binding assay.

**Compounds**	**Agonist potency (pEC_50_ ± SEM)**	**Intrinsic activity (E_max_ ± SEM in %)**
(+) 8-OH-DPAT	6.8 ± 0.2	94.0 ± 2.0
Buspirone	6.6 ± 0.3	33.0 ± 1.0
D1	6.1 ± 0.1	26.0 ± 2.0
WAY	0	0

The values of pEC_50_ (negative logarithm of the concentration of agonist required to elicit a response halfway between the baseline and maximum responses) and E_max,_ (maximal drug effect on [^35^S]GTPγS binding baseline expressed as a percentage of that produced by 10 μM (+)8-OH-DPAT) were estimated using nonlinear regression analysis.

**Table 2:** Report on behavioural alteration elicited by subcutaneous (s.c), intraperitoneal (i.p) or oral (p.o) administration of D1.

**Observation time after administration**	**Dose (mg/kg)**	**Administration routes and observations**		
		**s.c**	**i.p**	**p.o**
15 min	2, 10 or 50	N	N	N
	250	Reduced exploration	N	N
30 min	2 or 10	N	N	N
	50	Reduced exploration	Reduced exploration	Increase exploratory
	250	Sedation	Sedation	Increase exploratory
1 hr	2 or 10	Effects after 30 min of administrations persisted		
	50			
	250			
4 hr – 7 days	Total recovery from the effects of D1 without any sign of toxicity, weight gain or death occurence			

N - No observable behavioural alteration as compared to vehicle treated group.
